# Oblique lateral interbody fusion versus transforaminal lumbar interbody fusion for single-level lumbar adjacent segment disease: a retrospective comparative study of clinical and radiographic outcomes

**DOI:** 10.3389/fsurg.2026.1900293

**Published:** 2026-09-09

**Authors:** Kaiqiang Zhang, Li Xia, Zhenbin Wang

**Affiliations:** 1 The Fourth Clinical Medical College of Xinjiang Medical University, Urumgi, Xinjiang Uygur Autonomous Region, China; 2 Department of Second Surgery, Xinjiang Corps Hospital of Chinese People's Armed Police Force, Urumqi, Xinjiang, China; 3 The Fourth Affiliated Hospital of Xinjiang MedicaI University, Urumgi, Xinjiang Uygur Autonomous Region, China

**Keywords:** adjacent segment disease, oblique lateral lumbar interbody fusion, radiographic correction, spinal revision surgery, transforaminal lumbar interbody fusion

## Abstract

**Objective:**

To compare the clinical and radiographic outcomes of oblique lateral lumbar interbody fusion (OLIF) versus transforaminal lumbar interbody fusion (TLIF) for single-level lumbar adjacent segment disease (ASDis).

**Methods:**

A retrospective study was conducted on 107 patients who underwent revision surgery for single-level ASDis. Patients were divided into OLIF group (*n* = 55) and TLIF group (*n* = 52). Perioperative parameters (operative time, blood loss), clinical outcomes (VAS for low back and leg pain, Oswestry Disability Index, EQ-5D), radiographic parameters (disc height, segmental lordosis, segmental coronal angle, lumbar lordosis, PI-LL), complication and fusion rates were compared. Follow-up was ≥12 months.

**Results:**

OLIF had significantly longer operative time (308.5 ± 62.7 vs. 271.3 ± 55.9 min, *P* = 0.002) but less blood loss (362.4 ± 51.3 vs. 608.7 ± 76.5 mL, *P* < 0.001). No significant differences were found between groups in VAS, ODI, EQ-5D scores at any time point (*P* > 0.05). OLIF demonstrated superior correction of disc height (*^Δ^*3.9 ± 1.4 vs. 2.2 ± 0.8 mm, *P* < 0.001), segmental lordosis (*^Δ^*3.8°±1.7° vs. 2.3°±1.8°, *P* < 0.001), and segmental coronal angle (*^Δ^*1.6°±0.6° vs. 1.2°±0.5°, *P* < 0.001). No significant differences were observed in lumbar lordosis, PI-LL correction, complication rates (10.91% vs. 9.62%, *P* = 0.826), cage subsidence (12.73% vs. 11.54%, *P* = 0.850), reoperation rates (3.64% vs. 1.92%, *P* = 0.618), or fusion rates (92.73% vs. 92.31%, *P* = 0.934).

**Conclusion:**

OLIF and TLIF provide equivalent clinical outcomes for single-level lumbar ASDis. OLIF offers advantages in reducing intraoperative blood loss and restoring segmental radiographic parameters, at the cost of longer operative time. TLIF is associated with shorter surgery. Both techniques have comparable complication and fusion rates. Surgical choice should be individualized.

## 1 Introduction

Lumbar fusion is a classic surgical treatment for lumbar degenerative diseases; however, postoperative adjacent segment degeneration (ASD) has become a major long-term complication. When imaging evidence of degeneration is accompanied by corresponding clinical symptoms—such as new-onset low back pain and radicular pain in the lower limbs—the condition is defined as ASDis. The reported radiographic incidence of ASD ranges from 4.7% to 77%, while the clinical incidence of ASDis is approximately 5% to 30% (1, 2). A meta-analysis of 7,374 patients identified obesity, floating fusion, and facet joint invasion as significant risk factors for ASD (3). Furthermore, a meta-analysis further confirmed that advanced age, long segment fusion, and sagittal imbalance also increase the risk of ASD following posterior fusion (4).

For ASDis refractory to conservative treatment, surgical revision remains the only effective intervention. Although traditional posterior decompression with extended fusion provides reliable clinical outcomes, it is associated with several challenges: dissecting through scar tissue from the prior fusion carries a high risk of secondary paravertebral muscle injury and dural tear; moreover, extensive posterior dissection and facet resection further compromise the posterior structures, increasing blood loss and postoperative axial low back pain (5).

In recent years, OLIF has been introduced as a minimally invasive technique for ASDis revision. By traversing the natural retroperitoneal space, OLIF avoids paravertebral muscle dissection and allows the implantation of a large, anteriorly convex fusion cage, thereby better restoring intervertebral disc height and segmental lordosis (6, 7). Multiple meta-analyses have confirmed that OLIF offers significant advantages over TLIF in reducing intraoperative blood loss, shortening hospital stay, and improving disc height (7, 8). However, the incidence of cage subsidence after OLIF remains a concern; age >60 years, reduced bone mineral density, and a high cage profile have been identified as independent risk factors (9). Another widely performed procedure is TLIF, which accomplishes decompression and fusion through a unilateral posterior approach. TLIF benefits from high technical familiarity and is particularly suitable for patients with lateral approach constraints or retroperitoneal adhesions (10). The complication spectrum of TLIF mainly consists of dural tears and surgical site infections (11).

Currently, studies have compared the outcomes of OLIF and posterior lumbar interbody fusion (PLIF), yet direct comparisons between OLIF and TLIF remain limited. Chang et al. (12) demonstrated that OLIF was superior to TLIF in improving intervertebral disc height and segmental lordosis, while Liu et al. (10) reported that OLIF offers greater advantages in maintaining sagittal balance and slowing adjacent segment degeneration. A recently published meta-analysis further supports these findings, indicating that OLIF outperforms TLIF in terms of functional recovery and radiographic correction (7, 8). Nevertheless, systematic comparisons focusing on perioperative parameters, radiographic correction, and fusion rates specifically for single-segment ASDis are still lacking.

Therefore, this study retrospectively compared the clinical and radiographic outcomes of single-segment OLIF and TLIF for ASDis, analyzing the differences in operative time, blood loss, functional scores, and radiographic parameters between the two groups, with the aim of providing an evidence-based reference for individualized surgical decision-making.

## 2 Materials and methods

### 2.1 Research subject

This retrospective study included 107 patients who developed adjacent segment disease (ASDis) after lumbar fusion surgery and were admitted to the The Fourth Affiliated Hospital of Xinjiang Medical University between January 2019 and March 2024. The clinical data of these patients were retrospectively analyzed. Based on the surgical approach, the patients were divided into an oblique lumbar interbody fusion (OLIF) group (*n* = 55) and a transforaminal lumbar interbody fusion (TLIF) group (*n* = 52). The choice of surgical technique was determined by the surgeon's experience and the patient's specific lumbar anatomy. All patients in the OLIF group underwent extension of fixation using the original posterior pedicle screws. This study is a retrospective study that only collected routine clinical data from patients' prior diagnostic and treatment processes, involving no prospective intervention. This study involves no risk of personal privacy breach and has no commercial interests.This study was approved by the Ethics Committee of The Fourth Affiliated Hospital of Xinjiang Medical University.

### 2.2 Inclusion and exclusion criteria

#### 2.2.1 Inclusion criteria

(1) The primary surgery was lumbar fusion; follow-up MRI revealed ASD signs accompanied by concordant low back pain, radicular leg pain, or lower-extremity motor weakness, and the diagnosis of ASDis was confirmed by symptom relief following a diagnostic block.
(2) At least 6 weeks of conservative treatment had been completed without meaningful improvement or with symptom progression.
(3) The ASDis segment exhibited instability or spondylolisthesis, or decompression alone was deemed insufficient because of iatrogenic instability/slip risk; preoperative evaluation indicated the patient could tolerate decompression and fusion at that level.
(4) Radicular leg symptoms were prominent during standing and walking and relieved when lying flat, meeting the indications for OLIF.
(5) Postoperative follow-up duration was ≥12 months.

#### 2.2.2 Exclusion criteria

**Figure 1:**
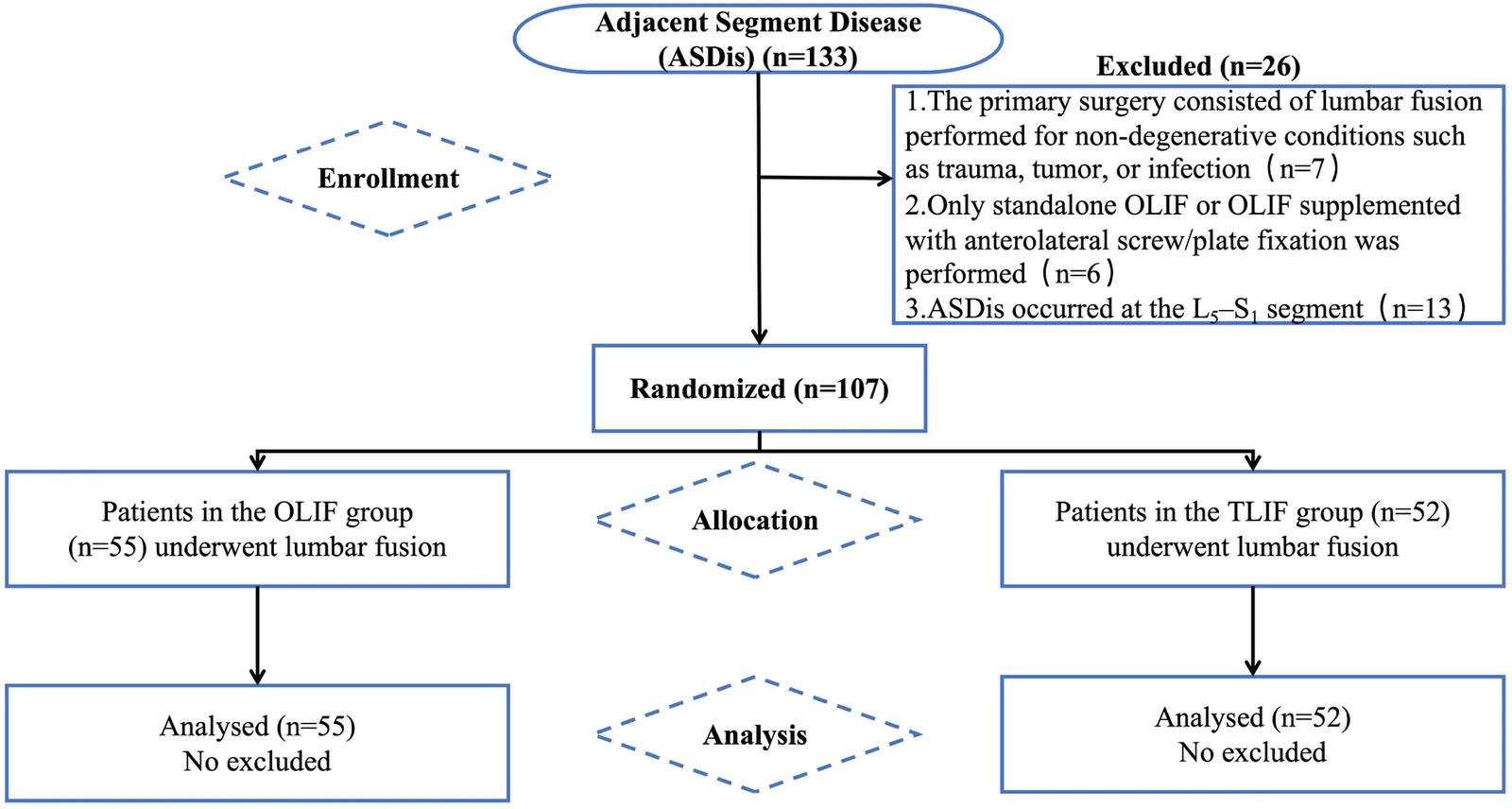
Process diagram for inclusion and exclusion criteria.

As shown in Figure 1.

(1) The primary surgery consisted of lumbar fusion performed for non-degenerative conditions such as trauma, tumor, or infection.
(2) Only standalone OLIF or OLIF supplemented with anterolateral screw/plate fixation was performed.
(3) ASDis occurred at the L_5_–S_1_ segment.

### 2.3 Surgical methods

#### 2.3.1 OLIF group

Under general anesthesia, the patient was placed in the right lateral decubitus position. Under fluoroscopic guidance, the center of the target disc, the lower endplate of the upper vertebral body, and the upper endplate of the lower vertebral body were marked. A 4 cm skin incision was made 5 cm anterior to the center of the target intervertebral disc, and the external oblique, internal oblique, and transversus abdominis muscles were separated layer by layer. The peritoneal contents were retracted anteriorly to expose the retroperitoneal space, and the psoas major muscle was retracted posteriorly to expose the intervertebral disc. After discectomy, a polyetheretherketone (PEEK) fusion cage with a 6° lordotic angle was implanted. The cage was packed with artificial bone graft; to enhance the fusion rate, autologous bone harvested from the vertebral margin or other sites could be mixed with the artificial bone for grafting. Once the cage position was confirmed fluoroscopically, the incision was closed. The patient was then repositioned to the prone position, and the skin incision was made along the previous surgical scar (via the midline or Wiltse approach) and extended. The posterior instrumentation was extended to the adjacent segment disease (ASD) level, and pedicle screws were inserted using an open technique. Any original screw that was loose or incompatible with the new rod was replaced with a larger-diameter screw. A drainage tube was placed through the posterior incision and was removed when the drainage volume was less than 50 mL over 2–3 days postoperatively. The patient wore a lumbar orthosis for 3 months after surgery.

#### 2.3.2 TLIF group

With the patient in the prone position, an incision was made along the original scar to expose the target segment and the previous implants. Partial facetectomy and laminectomy were performed to decompress the lateral recess and the neural foramen, thereby relieving compression of both the exiting and traversing nerve roots. After preparation of the intervertebral space, a single fusion cage packed with autologous bone or artificial bone graft was implanted into the disc space (autologous iliac bone graft was used in 23 cases and artificial bone graft in 17 cases). Pedicle screws were inserted using a standard open technique, and the management of the posterior instrumentation—including replacement of any loose or incompatible screws with larger-diameter screws—was identical to that in the OLIF group. A drainage tube was placed through the posterior incision and was removed according to the same criteria as in the OLIF group (drainage volume < 50 mL over 2–3 days postoperatively). The patient wore a lumbar orthosis for 3 months after surgery.

### 2.4 Observation indexes

#### 2.4.1 General information

Demographic and perioperative data were retrospectively collected from the medical records for all patients. Demographic data included age, sex, body mass index (BMI), American Society of Anesthesiologists (ASA) physical status classification, preoperative comorbidities, the number of ASDis segments, the interval from the index fusion surgery to revision surgery, the number of segments fused at the index surgery, and the duration of follow-up.

#### 2.4.2 Perioperative indicators and complications

Perioperative parameters included operative time, intraoperative blood loss, length of hospital stay, cage subsidence, perioperative complications, and reoperation rate. Intraoperative blood loss was calculated using the standard gravimetric and volumetric method: blood loss (mL) = total fluid volume in the suction canister (mL) + weight difference of soaked gauze and surgical drapes (g, converted to mL at 1 g ≈ 1 mL) – total irrigation fluid used during surgery (mL). Postoperative hospital stay was defined as the time from the date of surgery to the date of discharge. The discharge criteria were standardized for both groups: (1) stable vital signs for ≥24 h, (2) drainage volume < 50 mL over 2–3 days postoperatively with the drain removed, (3) ability to ambulate independently with a lumbar orthosis, (4) tolerance of oral intake without gastrointestinal symptoms, and (5) adequate pain control with oral analgesics. Reoperation was defined as any reoperation performed within one year after surgery for issues related to the revision procedure or adjacent segment disease.

#### 2.4.3 Efficacy index

The following clinical outcome measures were assessed preoperatively, at 3 months postoperatively, and at the last follow-up:

(1) Oswestry Disability Index (ODI) (13).
(2) EuroQol-5 Dimensions (EQ-5D) (14).
(3) Visual analog scale (VAS) for low back pain and leg pain (15).

All patients were followed up via outpatient visits or telephone interviews.

#### 2.4.4 Imaging indicators

The following radiographic parameters were measured on anteroposterior and lateral spine radiographs obtained preoperatively and at 1 year postoperatively for all patients:

(1) Disc height (DH): the average of the anterior, middle, and posterior intervertebral disc heights.
(2) Segmental lordosis (SL): the angle between the upper endplate of the upper vertebral body and the lower endplate of the lower vertebral body.
(3) Segmental coronal angle (SCA): the lateral bending angle between the upper and lower vertebral endplates.
(4) Lumbar lordosis (LL): the Cobb angle between the superior endplate of L_1_ and the superior endplate of S_1_.
(5) Pelvic incidence (PI) minus lumbar lordosis (PI-LL). PI was measured as the angle between a line from the midpoint of the S_1_ superior endplate to the center of the femoral head and a line perpendicular to the S_1_ superior endplate. PI-LL was calculated as the absolute difference between PI and LL.

At the last follow-up, the fusion status of the ASDis was evaluated using the Santos criteria (16). The Santos criteria were selected because they were specifically developed for radiographic assessment of interbody fusion using radiolucent (carbon fiber/PEEK) cages, which were used in all patients in both groups. The criteria provide a practical three-tier classification (nonunion, partial fusion, and solid fusion) based on motion, radiolucent lines, and bridging bone formation around the cage. Although the Bridwell system is also commonly used for interbody fusion grading, the Santos criteria more closely match the specific implant type (PEEK cage) and surgical context (interbody fusion) of our study. A diagnostic accuracy study comparing multiple fusion grading scales found no significant differences in clinical correlation between these systems.

In this study, fusion success was defined as achieving Grade II or Grade III fusion.

On lateral radiographs, fusion cage subsidence was graded according to the following criteria:

Grade 0 was defined as no subsidence, while Grades I through III were defined as subsidence of the fusion cage, Tables 1, 2.

**Table 1:** Grading of interbody fusion according to the santos criteria.

Grade	Criteria
I	Nonunion, with motion or radiolucent lines around the fusion cage.
II	Partial fusion, with no motion around the instrumentation but without definitive bridging bone formation within or around the fusion cage.
III	Solid fusion, with no motion or radiolucent lines around the instrumentation and with clear bridging bone formation within or around the fusion cage.

**Table 2:** Grading of fusion cage subsidence based on postoperative disc height loss.

Grade	Postoperative DH loss	Interpretation
0	< 25%	No subsidence
I	≥ 25% and < 50%	Subsidence
II	≥ 50% and < 75%	Subsidence
III	≥ 75%	Subsidence

### 2.5 Statistical methods

Statistical analysis was performed using *SPSS* 24.0 software (IBM Corp., Armonk, NY, USA). Categorical data are presented as n (%) and were compared using the *χ*^2^ test, corrected *χ*^2^ test, or Fisher's exact test, as appropriate. Ordinal data were compared using the rank-sum test. The Shapiro–Wilk test was used to assess the normality of continuous data. Continuous data that followed a normal distribution are expressed as mean ± standard deviation (x¯ ± s) and were compared using the independent-samples t-test; otherwise, data are expressed as M (*P*_25_, *P*_75_) and were compared using the Mann–Whitney U test. Clinical outcome measures assessed at multiple time points (preoperatively, at 3 months postoperatively, and at the last follow-up) were compared using repeated measures analysis of variance (ANOVA). Mauchly's test of sphericity was employed, and if the sphericity assumption was violated, the Greenhouse–Geisser correction was applied to adjust the degrees of freedom. All patients completed the required follow-up assessments, and thus there were no missing data for the clinical and radiographic outcome measures analyzed in this study. To control for Type I error due to multiple comparisons of the five independent radiographic parameters (DH, SL, SCA, LL, PI-LL), the Bonferroni correction was applied. A two-sided *P*-value of < 0.01 (0.05/5) was considered statistically significant for these radiographic comparisons. A two-sided *P* < 0.05 was considered statistically significant for all other analyses.

## 3 Results

### 3.1 Comparison of baseline data

The baseline demographic and clinical characteristics of the two groups including age, sex, body mass index (BMI), American Society of Anesthesiologists (ASA) physical status classification, smoking history, comorbidities (diabetes mellitus and cardiovascular disease), number of fused segments at the primary surgery, the specific level of ASDis, the location of ASDis (cephalad or caudal), the time interval from primary fusion to revision surgery, and the duration of follow-up are summarized in Table 3. Statistical comparisons showed no significant differences between the two groups for any of the above parameters (*P* > 0.05), indicating that the two groups were comparable at baseline.

**Table 3:** Comparison of baseline characteristics between the OLIF and TLIF groups [n(%), x¯ ± s].

Baseline characteristic	OLIF group (*n* = 55)	TLIF group (*n* = 52)	*t*/*χ^2^*/*Z*	*P*
Age (years)	68.5 ± 10.2	69.8 ± 11.0	*t* = −0.635	0.527
Sex			*χ^2^* = 0.104	0.747
Male	26 (47.3)	23 (44.2)		
Female	29 (52.7)	29 (55.8)		
BMI (kg/m^2^)	25.3 ± 3.5	25.8 ± 3.4	*t* = −0.748	0.456
ASA grade			*Z* = −0.424	0.672
I	2 (3.6)	3 (5.8)		
II	36 (65.5)	32 (61.5)		
III	17 (30.9)	17 (32.7)		
Smoking history	9 (16.4)	7 (13.5)	*χ^2^* = 0.179	0.672
Diabetes mellitus	14 (25.5)	12 (23.1)	*χ^2^* = 0.084	0.772
Cardiovascular disease	10 (18.2)	13 (25.0)	*χ^2^* = 0.726	0.394
No. of primary fused segments			*χ^2^* = 0.276	0.871
Single level	21 (38.2)	19 (36.5)		
Two levels	26 (47.3)	24 (46.2)		
Three levels	8 (14.5)	9 (17.3)		
ASDis level			*χ^2^* = 0.941	0.918
L_1–2_	3 (5.5)	5 (9.6)		
L_2–3_	12 (21.8)	10 (19.2)		
L_3–4_	24 (43.6)	22 (42.3)		
L_4–5_	16 (29.1)	15 (28.9)		
ASDis location			*χ^2^* = 0.039	0.844
Cephalad	39 (70.9)	36 (69.2)		
Caudal	16 (29.1)	16 (30.8)		
Time from primary fusion to revision (years)	6.5 ± 2.8	6.2 ± 2.6	*t* = 0.572	0.568
Follow-up duration (months)	18.9 ± 3.6	19.3 ± 3.8	*t* = −0.558	0.578

Continuous variables were compared using independent-sample t test; categorical variables were analyzed using χ^2^test; ordinal data (ASA grade) were analyzed using Mann-Whitney U test. *P* < 0.05 was considered statistically significant.

### 3.2 Comparison of perioperative outcomes and complications

Revision surgery was successfully completed in all patients in both groups, with no intraoperative conversions to an alternative procedure or aborted surgeries. Perioperative outcome parameters and complications are summarized in Table 4. Compared with the TLIF group, the OLIF group was associated with a significantly longer operative time (*P* = 0.002) but markedly less intraoperative blood loss (*P* < 0.001). No significant differences were observed between the two groups regarding postoperative hospital stay, cage subsidence rate, overall perioperative complication rate, or reoperation rate (*P* > 0.05).

**Table 4:** Comparison of perioperative outcomes between the OLIF and TLIF groups [n(%), x¯ ± s].

Indicator	OLIF group (*n* = 55)	TLIF group (*n* = 52)	*t/χ^2^*	*P*
Operative time (min)	308.5 ± 62.7	271.3 ± 55.9	*t* = 3.241	0.002
Intraoperative blood loss (mL)	362.4 ± 51.3	608.7 ± 76.5	*t* = −19.627	<0.001
Postoperative hospital stay (days)	6.7 ± 2.4	6.9 ± 1.8	*t* = −0.484	0.629
Cage subsidence	7 (12.73)	6 (11.54)	*χ^2^* = 0.036	0.850
Perioperative complications	6 (10.91)	5 (9.62)	*χ^2^* = 0.049	0.826
Reoperation rate	2 (3.64)	1 (1.92)	*χ^2^* = 0.249	0.618

Continuous variables were compared using the independent-samples t-test; categorical variables were analyzed with the *χ^2^* test (or Fisher's exact test when the expected frequency in any cell was <5). Intraoperative blood loss was calculated using the gravimetric and volumetric method (suction canister volume + gauze/drape weight difference – irrigation fluid volume). Postoperative hospital stay was defined as the time from surgery to discharge, with standardized discharge criteria applied to both groups.

In the OLIF group, six patients (10.91%) experienced perioperative complications: three cases of ileus, two cases of transient lower limb pain or numbness, and one superficial incisional infection. All resolved with symptomatic treatment. In the TLIF group, five patients (9.62%) developed complications: two dural tears (which were successfully repaired intraoperatively), one ileus, one deep surgical site infection, and one case of postoperative radicular pain that resolved with conservative management. The spectrum of complications in both groups was consistent with that reported in the existing literature.

Two patients (3.64%) in the OLIF group required reoperation: one for progression of adjacent segment disease warranting an additional revision fusion, and one for pedicle screw breakage necessitating hardware revision. In the TLIF group, one patient (1.92%) underwent reoperation for progression of adjacent segment disease. The reoperation rates did not differ significantly between the two groups (*P* = 0.618).

### 3.3 Efficacy outcomes

**Figure 2:**
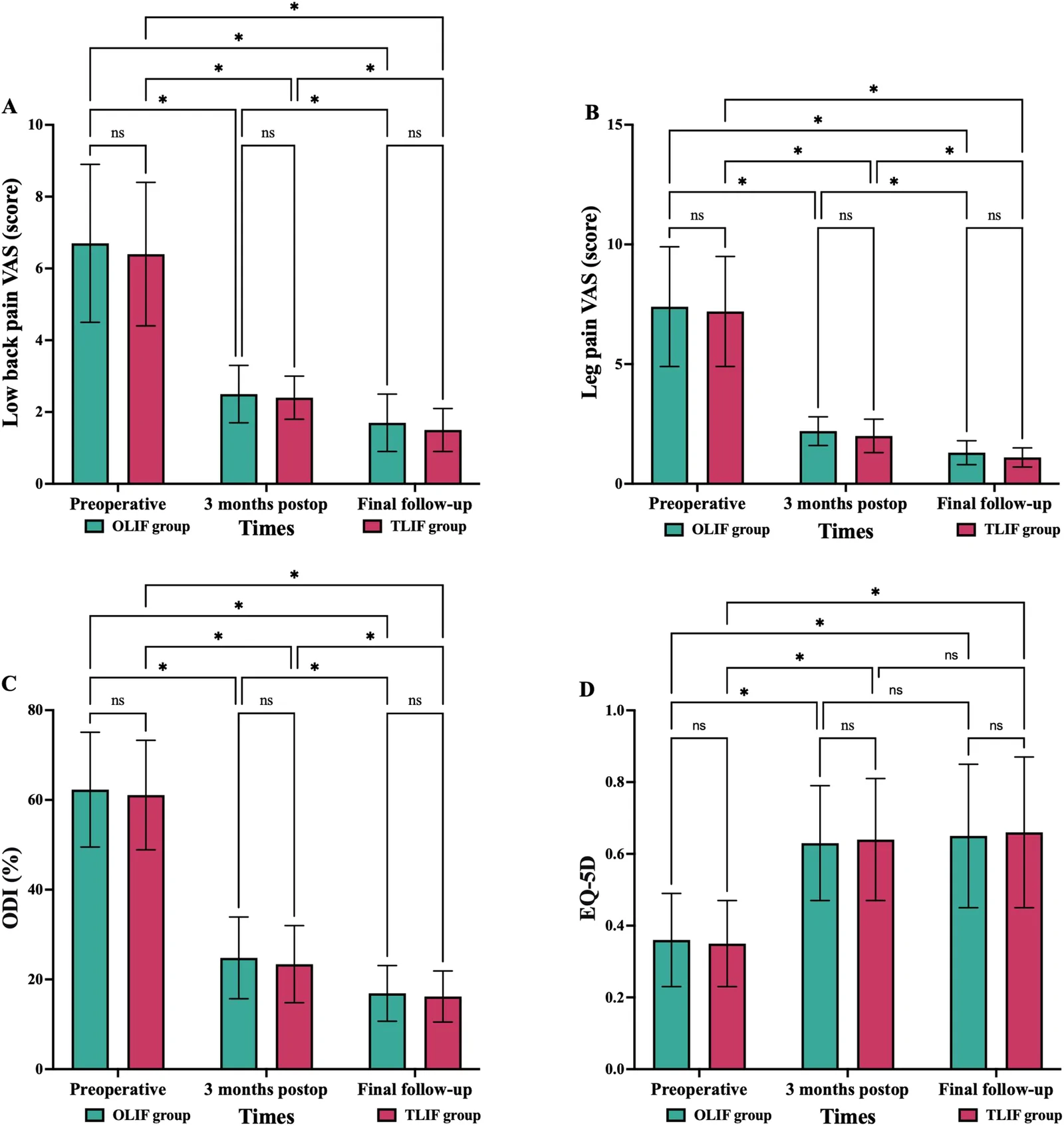
Comparison of low back pain VAS scores between OLIF and TLIF groups at preoperative, 3-month postoperative, and final follow-up. **(A)** Low back pain VAS. **(B)** Leg pain VAS. **(C)** Oswestry disability index (ODI). D, EQ-5D. Data are presented as mean ± SD. *, *P* < 0.001 compared with preoperative values. ns, no significant between-group differences were observed at any time point (*P* > 0.05).

Preoperative, 3-month postoperative, and final follow-up scores for low back pain VAS, leg pain VAS, ODI, and EQ-5D are summarized in Figure 2. Repeated-measures ANOVA with Greenhouse–Geisser correction when necessary showed no significant main effect of group for any outcome (*P* > 0.05), a significant main effect of time (*P* < 0.001), and no significant group-by-time interaction (*P* > 0.05). These findings indicate that the two surgical techniques provide comparable pain relief, functional improvement, and gains in quality of life.

The mean improvements from baseline to final follow-up in the OLIF group were 3.4 for low back pain VAS, 3.6 for leg pain VAS, 21.3 for ODI, and 0.24 for EQ-5D; the corresponding values in the TLIF group were 3.2, 3.3, 20.1, and 0.22, respectively. All of these improvements exceeded the established MCID thresholds for lumbar fusion surgery (VAS back pain: 2.1–3.0 points; VAS leg pain: 2.4–2.8 points; ODI: 10–15 points; EQ-5D: 0.19–0.28).

**Figure 3:**
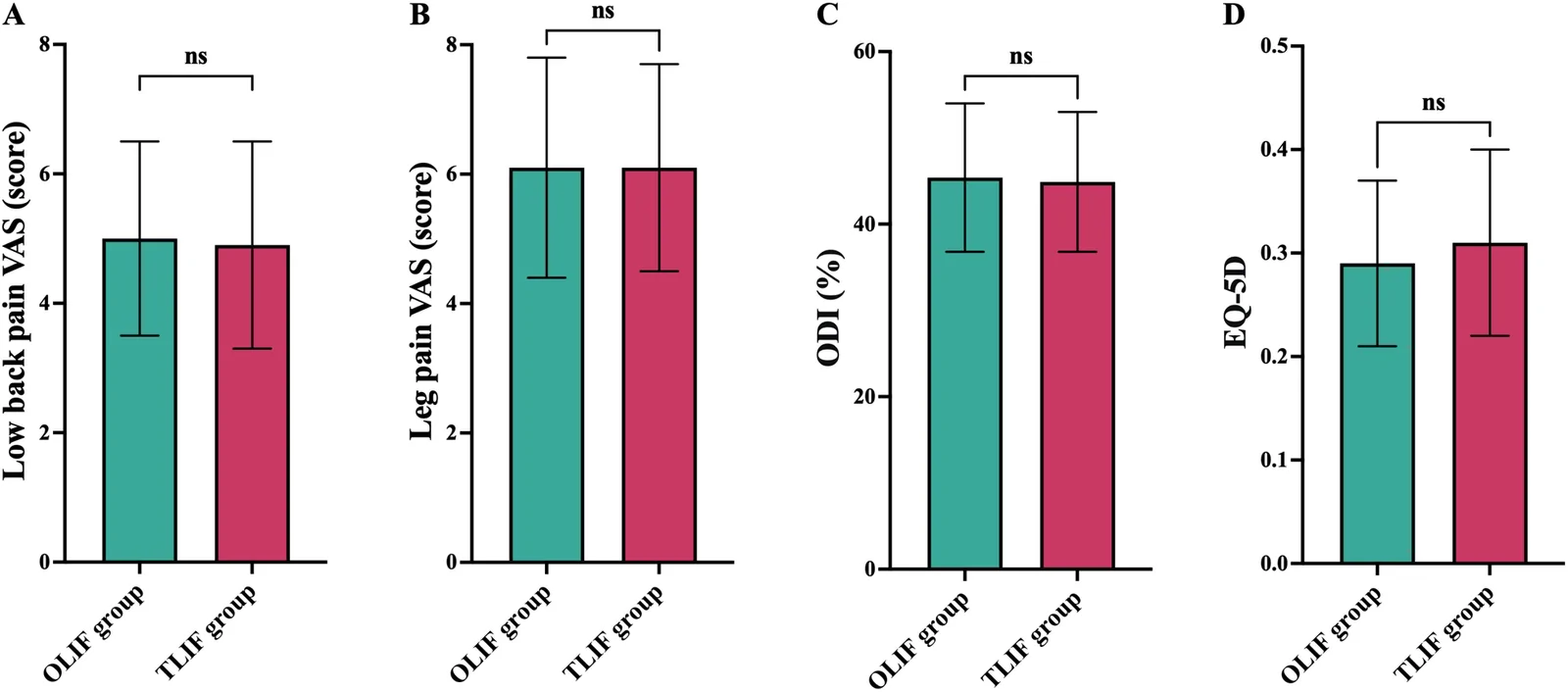
Comparison of improvement in efficacy outcomes between the OLIF and TLIF groups. **(A)** Low back pain VAS. **(B)** Leg pain VAS. **(C)** Oswestry disability index (ODI). **(D)** EQ-5D. Data are presented as mean ± SD. ns, no significant between-group differences were observed (*P* > 0.05).

The absolute improvements from preoperative to final follow-up for each efficacy outcome are compared between the groups in Figure 3. No significant between-group differences were observed (*P* > 0.05).

### 3.4 Radiological outcomes

Preoperative and 1-year postoperative radiographic parameters disc height (DH), segmental lordosis (SL), segmental coronal angle (SCA), lumbar lordosis (LL), and PI-LL are summarized in Table 5. The groups did not differ significantly on any preoperative parameter (*P* > 0.05). At the 1-year follow-up, the OLIF group achieved significantly greater correction of DH, SL, and SCA than the TLIF group, with all *P*-values remaining below the Bonferroni-corrected significance threshold of *P* < 0.01 (*P* < 0.001, *P* < 0.001, and *P* < 0.001, respectively). In contrast, no significant between-group differences were detected in the correction of LL, correction of PI-LL, or the fusion rate (*P* > 0.05).

**Table 5:** Comparison of radiological outcomes between the OLIF and TLIF groups (x¯ ± s).

Radiological parameter	Time point	OLIF group (*n* = 55)	TLIF group (*n* = 52)	*t*/Fisher	*P*
DH (mm)	Preoperative	5.0 ± 1.3	5.1 ± 1.4	−0.382	0.703
	1-year postop	8.9 ± 1.8*	7.3 ± 1.5*	4.970	<0.001
	Correction^a^	3.9 ± 1.4	2.2 ± 0.8	7.869	<0.001
SL (°)	Preoperative	4.8 ± 1.5	5.1 ± 1.6	−1.008	0.316
	1-year postop	8.6 ± 2.0*	7.4 ± 2.3*	2.856	0.005
	Correction^a^	3.8 ± 1.7	2.3 ± 1.8	4.412	<0.001
SCA (°)	Preoperative	2.8 ± 1.3	2.7 ± 1.2	0.409	0.683
	1-year postop	1.2 ± 0.5*	1.5 ± 0.6*	−2.867	0.005
	Correction^a^	1.6 ± 0.6	1.2 ± 0.5	3.724	<0.001
LL (°)	Preoperative	45.9 ± 11.5	44.1 ± 10.9	0.835	0.405
	1-year postop	47.2 ± 12.0	45.3 ± 11.5	0.836	0.405
	Correction^a^	1.3 ± 1.1	1.2 ± 1.0	0.486	0.628
PI-LL (°)	Preoperative	14.5 ± 4.9	13.9 ± 4.6	0.648	0.518
	1-year postop	12.3 ± 4.4	12.6 ± 4.2	−0.364	0.717
	Correction^a^	2.2 ± 0.9	1.9 ± 0.8	1.801	0.074
Fusion rate (n, %)	Final follow-up	51 (92.73)	48 (92.31)	–	0.934

*P < 0.05 compared with preoperative value.

a Correction is defined as the absolute difference between the 1-year postoperative value and the preoperative value. For comparisons of radiographic parameters (DH, SL, SCA, LL, PI-LL), statistical significance was set at *P* < 0.01 after Bonferroni correction. Fusion rate comparison was performed using Fisher's exact test.

At final follow-up, fusion status was evaluated using the Santos criteria. Grade II or III fusion was attained in 51 patients (92.73%) in the OLIF group and in 48 patients (92.31%) in the TLIF group, a difference that was not statistically significant (*P* = 0.934, Fisher's exact test).

### 3.5 Typical cases

#### 3.5.1 Case 1: TLIF group

**Figure 4:**
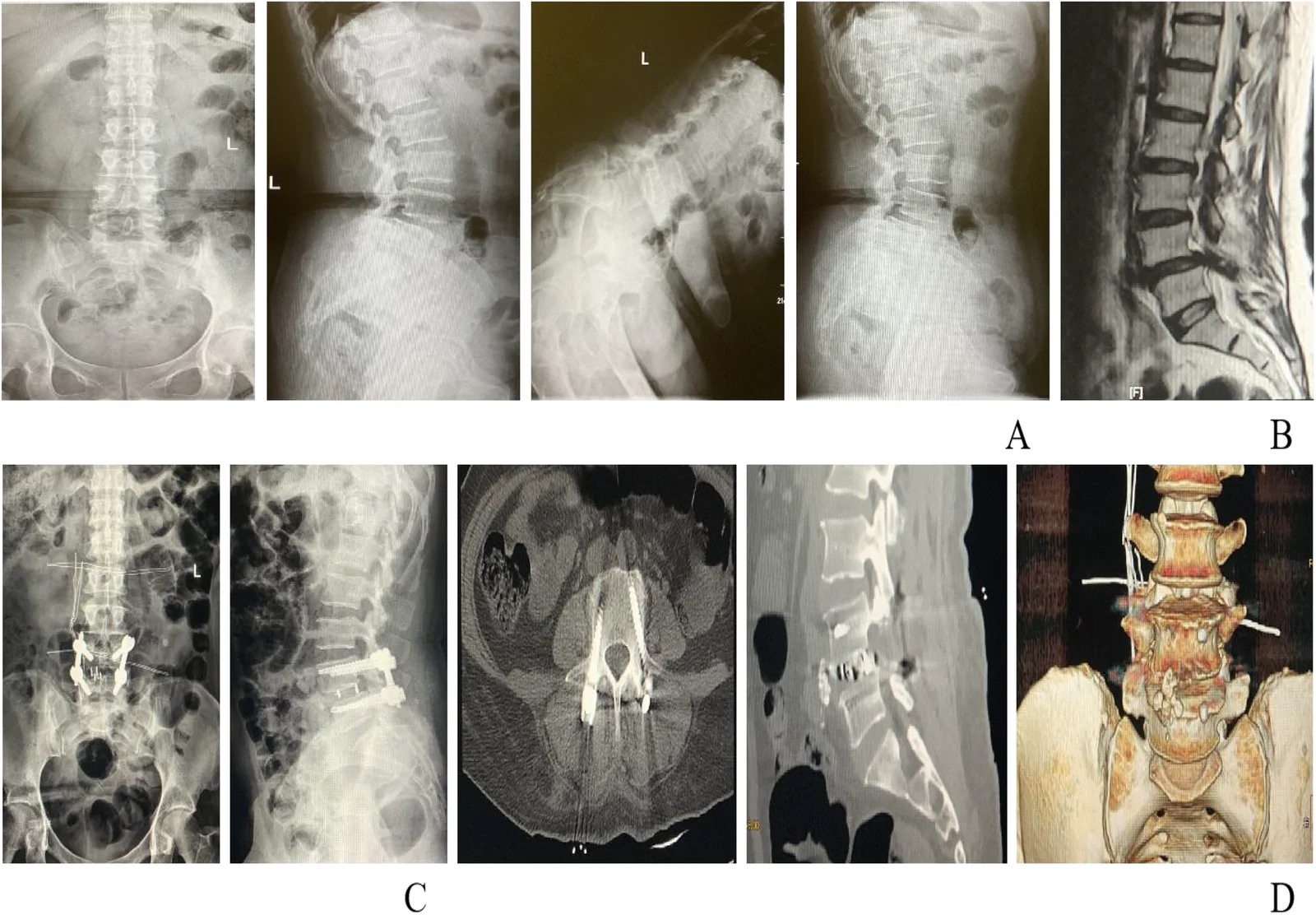
Representative case in the TLIF group.

A patient presented with bilateral lower-limb pain for 5 months, aggravated by low back pain for 2 days. The preoperative diagnosis was lumbar spondylolisthesis combined with lumbar disc herniation, and the patient underwent transforaminal lumbar interbody fusion. Figure 4A, preoperative lumbar radiographs showed degenerative spondylolisthesis and segmental instability at L_4−5_, with narrowing of the intervertebral disc space. Figure 4B, preoperative sagittal MRI demonstrated L_4−5_ disc degeneration/herniation with compression of the dural sac and lumbar spinal canal stenosis. Figure 4C, postoperative anteroposterior and lateral radiographs showed satisfactory placement of the interbody cage and bilateral pedicle screw-rod fixation, with improved disc height and segmental alignment. Figure 4D, postoperative CT images and three-dimensional reconstruction confirmed appropriate positions of the cage and pedicle screws, without obvious cage migration, screw malposition, or implant loosening.

#### 3.5.2 Case 2: OLIF group

**Figure 5:**
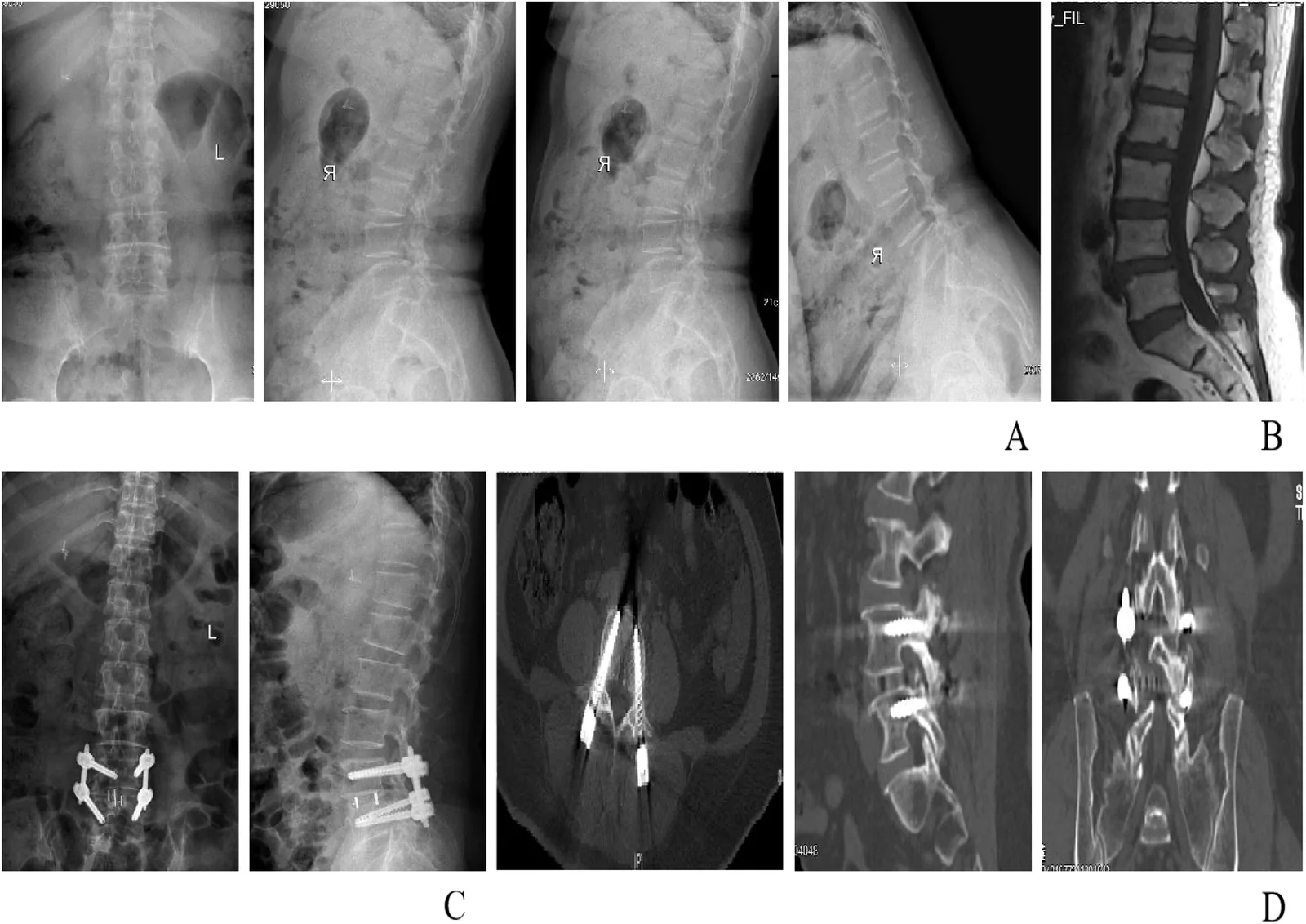
Representative case in the OLIF group.

A patient presented with low back pain associated with right lower-limb pain for 1 year. The preoperative diagnosis was lumbar spondylolisthesis combined with lumbar instability, and the patient underwent oblique lateral lumbar interbody fusion. Figure 5A, preoperative lumbar radiographs showed degenerative lumbar spondylolisthesis and segmental instability, with narrowing of the intervertebral disc space. Figure 5B, preoperative sagittal MRI demonstrated lumbar disc degeneration with compression of the dural sac and lumbar spinal canal stenosis. Figure 5C, postoperative anteroposterior and lateral radiographs showed satisfactory placement of the interbody cage and posterior pedicle screw-rod fixation, with restoration of disc height and improvement of segmental alignment. Figure 5D, postoperative CT images and three-dimensional reconstruction confirmed appropriate positions of the cage and pedicle screws, without obvious cage migration, screw malposition, or implant loosening.

## 4 Discussion

In this retrospective comparative study of 107 patients who underwent revision surgery for single-level lumbar adjacent segment disease (ASDis), oblique lateral lumbar interbody fusion (OLIF) and transforaminal lumbar interbody fusion (TLIF) achieved equivalent clinical outcomes with respect to pain relief, functional recovery, and health-related quality of life. Nonetheless, OLIF was associated with significantly lower intraoperative blood loss and superior radiographic correction of disc height (DH), segmental lordosis (SL), and segmental coronal angle (SCA), at the expense of a longer operative time. Perioperative complication rates, cage subsidence rates, and reoperation rates did not differ significantly between the groups, and both techniques yielded similarly high fusion rates (OLIF 92.73% vs. TLIF 92.31%). These findings provide an evidence-based reference for individualizing the surgical approach in patients with single-level ASDis.

The absence of any significant differences in low back pain VAS, leg pain VAS, ODI, and EQ-5D scores between the two groups at all follow-up time points (*P* > 0.05) confirms the clinical equivalence of OLIF and TLIF. More importantly, both groups achieved mean improvements that met or exceeded the established MCID thresholds: for VAS back pain (MCID: 2.1–3.0 points), VAS leg pain (MCID: 2.4–2.8 points), ODI (MCID: 10–15 points), and EQ-5D (MCID: 0.19–0.28). These findings indicate that both OLIF and TLIF provide clinically meaningful improvements in pain relief, functional recovery, and quality of life for patients with single-level ASDis. This result aligns with the meta-analysis by Xiao et al., who reported that OLIF and TLIF produced comparable improvements in VAS and ODI scores for lumbar degenerative diseases (7). Similarly, a comparative study by Chang et al. found no significant between-group differences in patient-reported outcomes at 12 months after surgery, despite differences in radiographic parameters (12). In a comprehensive systematic review and meta-analysis comparing transforaminal and lateral lumbar interbody fusion, Perez-Albela et al. observed that most patient-reported outcomes were comparable between the two approaches, further supporting the clinical equivalence demonstrated in our study (17). It is noteworthy that OLIF achieves neural decompression indirectly by restoring DH and foraminal dimensions, whereas TLIF accomplishes direct decompression through laminectomy and facetectomy. Our data indicate that indirect decompression via DH restoration (from 5.0 ± 1.3 mm preoperatively to 8.9 ± 1.8 mm postoperatively in the OLIF group) is sufficient to achieve clinical results comparable to those of direct decompression. This is in line with the findings of Avdeev et al., who reported that OLIF-based indirect decompression significantly increased the cross-sectional area of the vertebral canal and the depth of the lateral recess, with critical thresholds for successful decompression being a postoperative canal area ≥ 80 mm^2^ and a lateral recess depth ≥ 3 mm (16). It must be emphasized, however, that OLIF may not be appropriate for patients with severe central stenosis or large disc fragments that extend beyond the central canal, as indirect decompression has inherent limitations in such cases (5). Notably, a study specifically investigating MCID following revision surgery for ASDis reported thresholds of 3.8 points for back pain VAS, 2.4 points for leg pain VAS, and 6.8 points for ODI. The improvements observed in both our OLIF and TLIF groups exceeded these ASDis revision-specific thresholds as well, further supporting the clinical effectiveness of both procedures in this challenging patient population.

The most consistent radiographic advantage of OLIF over TLIF in our series was the greater improvement in DH (*^Δ^* 3.9 ± 1.4 mm vs. 2.2 ± 0.8 mm, *P* < 0.001), SL (*^Δ^* 3.8^°^ ± 1.7^°^ vs. 2.3^°^ ± 1.8^°^, *P* < 0.001), and SCA (*^Δ^* 1.6^°^ ± 0.6^°^ vs. 1.2^°^± 0.5^°^, *P* < 0.001). These findings agree well with those reported by Chang et al., who demonstrated that OLIF was associated with significantly better restoration of DH and SL compared with TLIF in a cohort of 65 patients with lumbar ASDis (12). The mechanical explanation for this advantage lies in the design and placement of the interbody cage. OLIF permits the insertion of a larger cage with a built-in 6^°^ lordotic angle through the anterior column, whereas TLIF is constrained by the need to pass the cage through the intervertebral foramen, which typically limits the cage to a smaller, non-lordotic design. A computed tomography-based analysis by Foong et al. showed that OLIF facilitates appropriate cage placement with only a minor degree of cage obliquity (mean 4.2^°^ ± 2.8^°^) and that this minor obliquity does not compromise fusion rates or lead to increased subsidence or sagittal malalignment (18). In a large systematic review and meta-analysis comparing multiple lumbar interbody fusion techniques, Lechtholz-Zey et al. demonstrated that OLIF provided superior restoration of both segmental and global lumbar lordosis compared with TLIF, with the meta-analysis confirming that TLIF conferred significantly lower SL restoration than OLIF (*P* < 0.05) (19). This high-level evidence strongly corroborates our radiographic findings. Nevertheless, the superior segmental correction achieved with OLIF did not translate into significant differences in global lumbar lordosis (*^Δ^* 1.3^°^ ± 1.1^°^ vs. 1.2^°^ ± 1.0^°^, *P* = 0.628) or PI-LL mismatch (*^Δ^* 2.2^°^ ± 0.9^°^ vs. 1.9^°^ ± 0.8^°^, *P* = 0.074) between the groups. This suggests that, in single-level ASDis revisions, local radiographic correction does not automatically influence global sagittal balance, an observation that has also been noted in other studies (19).

Although the differences in DH, SL, and SCA correction between the two groups were statistically significant, the minimal clinically important difference for these radiographic parameters in the context of ASDis revision surgery has not been firmly established. However, several studies have attempted to link radiographic restoration to clinically meaningful outcomes. For instance, a study on indirect decompression efficacy in OLIF suggested that a DH correction of 2.4 mm may serve as a predictive threshold for successful canal decompression. In our OLIF cohort, the mean DH correction (3.9 ± 1.4 mm) substantially exceeded this proposed threshold, whereas the TLIF cohort (2.2 ± 0.8 mm) did not. Similarly, changes in segmental lordosis have been identified as critical parameters for achieving MCID in patient-reported outcomes following lumbar fusion surgery. These observations suggest that the superior radiographic restoration achieved with OLIF may translate into clinically relevant biomechanical benefits, although direct evidence linking specific radiographic MCID thresholds to patient-reported outcomes in ASDis revision surgery remains limited and warrants further investigation.

Regarding perioperative parameters, our study confirmed that OLIF was associated with significantly less intraoperative blood loss (362.4 ± 51.3 mL vs. 608.7 ± 76.5 mL, *P* < 0.001) but a longer operative time (308.5 ± 62.7 min vs. 271.3 ± 55.9 min, *P* = 0.002). These results are consistent with those of Chang et al., who reported operative times of 283.1 ± 59.6 min for OLIF and 254.1 ± 55.8 min for TLIF, and blood loss of 371.9 ± 257.4 mL vs. 622.0 ± 213.8 mL, respectively (12). A multicenter randomized controlled trial comparing OLIF with lateral vertebral screw fixation vs. TLIF for severe lumbar stenosis found that OLIF was associated with shorter operative time and less blood loss (20). It should be noted that the operative time findings in that trial differ from ours, which may be attributable to differences in surgical protocols specifically, our two-stage OLIF procedure required patient repositioning, whereas the lateral screw fixation technique eliminates the need for a separate posterior fixation step. In a large meta-analysis of 24 studies comprising 1,785 patients, Liu et al. compared minimally invasive TLIF with OLIF for lumbar degenerative diseases and concluded that OLIF was associated with significantly lower intraoperative blood loss, shorter hospital stays, and shorter operative time compared with MIS-TLIF (8). The longer operative time for OLIF in our series can be attributed to the two-stage procedure: after completing the anterior OLIF approach, patients were repositioned from the lateral decubitus to the prone position, followed by a second surgical preparation and draping for posterior pedicle screw fixation. The posterior fixation step was identical in both groups. Had OLIF been performed as a single-position procedure, the operative time might have been reduced. Recent evidence from Park et al. suggests that prone-position OLIF offers significantly shorter operative times compared with conventional OLIF while maintaining similar radiographic and clinical outcomes (21). The lower blood loss with OLIF is largely explained by the avoidance of posterior soft-tissue dissection and bone resection. TLIF requires extensive dissection of paraspinal muscles, removal of scar tissue from the prior fusion, facetectomy, and laminectomy, each of which contributes to increased bleeding. Zhu et al. similarly reported that OLIF was associated with significantly less blood loss than posterior lumbar interbody fusion in revision surgery for ASDis (10).

The overall complication rate was similar between the two groups (OLIF 10.91% vs. TLIF 9.62%, *P* = 0.826). This finding is consistent with a meta-analysis by Shi et al. comparing OLIF and TLIF for degenerative lumbar spondylolisthesis, which included 14 studies with 877 patients and found no significant difference in surgical complication rates between the two approaches (*P* > 0.05) (22). However, the types of complications differed in a manner that reflects the inherent risks of each surgical approach. In the OLIF group, three cases of postoperative ileus were observed. This complication is a direct consequence of the retroperitoneal approach: manipulation of the peritoneal contents and dissection through the retroperitoneal fat can cause mechanical irritation of the mesentery and postoperative bowel hypomotility. The incidence of ileus after OLIF has been reported to be around 2%–5% in large series, with advanced age, diabetes, and a history of constipation identified as risk factors (23). In the TLIF group, dural tears occurred in two patients (3.8%). This is consistent with the existing literature on TLIF complications; a large single-center study of 531 TLIF procedures by Tormenti et al. reported a durotomy rate of 14.3% in patients undergoing revision surgery, with revision surgery being a significant risk factor for incidental durotomy (24). The presence of scar tissue from the primary fusion makes dissection of the lamina and ligamentum flavum considerably more difficult. From a clinical decision-making perspective, the choice between OLIF and TLIF should take into account not only the overall complication rates but also the specific comorbidity profile of the patient. For patients with a history of abdominal surgery or a high risk of postoperative ileus, TLIF may be preferable. For patients with extensive posterior scar tissue from previous surgery, OLIF offers the advantage of avoiding the scarred posterior plane altogether.

Fusion rates were similarly high in both groups (92.73% for OLIF vs. 92.31% for TLIF, *P* = 0.934). The Santos criteria were used to assess fusion status, as they were specifically developed for interbody fusion using radiolucent cages (PEEK), which were implanted in all patients. While the Bridwell system is another widely used grading scale, a comparative diagnostic accuracy study demonstrated that different fusion grading systems, including Bridwell and Santos, show no significant differences in clinical correlation. Therefore, our fusion rate findings are robust and unlikely to be affected by the choice of grading system. This finding is in agreement with the meta-analysis by Xiao et al., which found no significant differences in fusion rates between OLIF and TLIF for lumbar degenerative diseases (7). A retrospective cohort study evaluating lateral fusion features reported fusion rates of 100% for OLIF stand-alone, 97.0% for OLIF with posterior screw fixation, and 96.4% for TLIF, with no significant differences between groups (*P* = 0.167), further supporting our results (25). In our OLIF group, cage subsidence (Grade I or higher) occurred in 12.73% of patients. This rate is similar to the 14.4% Grade I subsidence rate reported by Foong et al. in a series of 97 OLIF-treated levels (18). Importantly, despite subsidence being relatively common, the majority of these cases still achieved solid fusion. Park et al. recently evaluated the effects of the vacuum phenomenon on cage subsidence in OLIF and found that high vacuum grades significantly increased the risk of subsidence, but no significant differences in clinical outcomes or final fusion rates were observed across different vacuum grades (23). In the TLIF group, the use of autologous iliac bone graft (23 patients) yielded a fusion rate of 95.65% compared with 88.24% for artificial bone graft (*P* = 0.565). Although this difference did not reach statistical significance, likely due to the small sample size, it suggests that for patients at high risk of non-union, autologous bone graft may still be considered to optimize fusion outcomes.

Several limitations of this study must be acknowledged. First, this is a retrospective, single-center study, and selection bias cannot be completely eliminated despite the demonstration of baseline comparability between the two groups. Second, the mean follow-up of approximately 19 months is relatively short; longer follow-up is needed to assess the durability of the radiographic correction and to determine whether the superior segmental radiographic gains achieved with OLIF ultimately translate into a lower rate of recurrent ASDis or a reduced need for further reoperation. Third, the severity of spinal stenosis before surgery was not stratified, and the efficacy of indirect decompression by OLIF may differ between patients with mild versus severe preoperative canal compromise (5). Fourth, OLIF is not suitable for patients with severe central or foraminal stenosis that requires direct decompression, a contraindication that should be respected. Fifth, the conclusions may not be generalizable to multisegment ASDis revisions, which involve a different risk-benefit balance. Finally, all patients in this study were older adults, and the applicability of these findings to younger, more active patients requires further investigation.

In conclusion, both OLIF and TLIF are effective surgical options for single-level lumbar ASDis, with comparable clinical outcomes in terms of pain relief, functional recovery, and quality of life. OLIF offers the advantages of less intraoperative blood loss and superior restoration of disc height, segmental lordosis, and coronal alignment, at the cost of a longer operative time. TLIF is associated with shorter operative times but greater blood loss and a complication profile dominated by dural tears. The choice between the two techniques should be individualized based on patient anatomy, comorbidity burden, and surgeon experience. Prospective studies with larger sample sizes and longer follow-up are warranted to confirm these findings and to further refine patient selection criteria.

## Data Availability

The raw data supporting the conclusions of this article will be made available by the authors, without undue reservation.
